# Emerging roles of SIRT1 activator, SRT2104, in disease treatment

**DOI:** 10.1038/s41598-024-55923-8

**Published:** 2024-03-06

**Authors:** Ning Chang, Junyang Li, Sufen Lin, Jinfeng Zhang, Weiqiang Zeng, Guoda Ma, Yajun Wang

**Affiliations:** Shunde Women and Children’s Hospital, Guangdong Medical University, Foshan, China

**Keywords:** SRT2104, SIRT1, Disease treatment, Preclinical research, Clinical trials, Drug discovery, Diseases

## Abstract

Silent information regulator 1 (SIRT1) is a NAD^+^-dependent class III deacetylase that plays important roles in the pathogenesis of numerous diseases, positioning it as a prime candidate for therapeutic intervention. Among its modulators, SRT2104 emerges as the most specific small molecule activator of SIRT1, currently advancing into the clinical translation phase. The primary objective of this review is to evaluate the emerging roles of SRT2104, and to explore its potential as a therapeutic agent in various diseases. In the present review, we systematically summarized the findings from an extensive array of literature sources including the progress of its application in disease treatment and its potential molecular mechanisms by reviewing the literature published in databases such as PubMed, Web of Science, and the World Health Organization International Clinical Trials Registry Platform. We focuses on the strides made in employing SRT2104 for disease treatment, elucidating its potential molecular underpinnings based on preclinical and clinical research data. The findings reveal that SRT2104, as a potent SIRT1 activator, holds considerable therapeutic potential, particularly in modulating metabolic and longevity-related pathways. This review establishes SRT2104 as a leading SIRT1 activator with significant therapeutic promise.

## Introduction

Silent information regulator 1 (SIRT1), a prominent member of the Sirtuin family, has been the subject of extensive research due to its crucial role in cellular processes. As a sensor of cellular energy metabolism changes, SIRT1 detects fluctuations in Nicotinamide Adenine Dinucleotide (NAD^+^) levels and translates these into epigenetic signals by altering the acetylation levels of its numerous substrates^1^. This dynamic process allows SIRT1 to exert a variety of functions, depending on its interactions with different substrates. Notably, SIRT1 regulates over 70 substrates, including key proteins such as tumor protein p53 (p53), peroxisome proliferator-activated receptor gamma coactivator 1-alpha (PGC-1α), nuclear factor kappa-light-chain-enhancer of activated B cells (NF-κB), Forkhead Box O (FOXO), nuclear receptor corepressor (NCoR), and E1A binding protein p300 (p300)^2–9^. Through these interactions, SIRT1 is intricately involved in regulating a multitude of cellular functions and physiological processes. These include critical areas like cell cycle control, energy expenditure, response to oxidative stress, cell apoptosis, and aging^10–15^. Furthermore, research has shown that calorie restriction (CR) leads to an increase in SIRT1 expression, which in turn has been linked to the extension of lifespan in mammals^16^.This fascinating discovery has ignited significant interest among researchers, prompting in-depth studies into the mechanisms of SIRT1 and its activators and their potential implications for human health and longevity.

## Evolution of SIRT1 activators: from resveratrol to SRT2104

**Figure 1:**
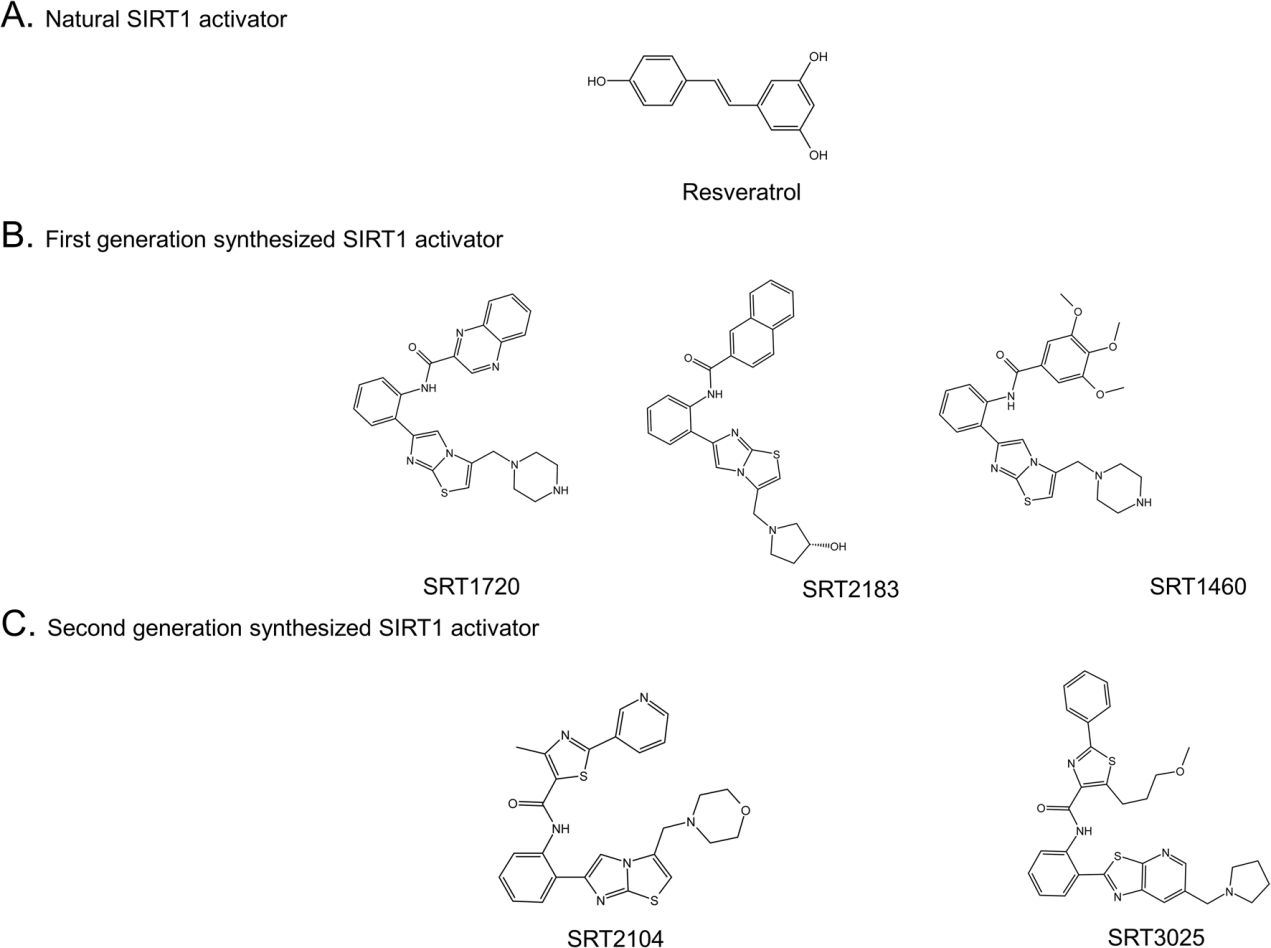
Chemical structure of a classical SIRT1 activator. (**A**) Natural SIRT1 activator. (**B**) First generation synthesized SIRT1 activator. (**C**) Second generation synthesized SIRT1 activator.

Resveratrol was the pioneering compound found to mimic the effects of calorie restriction by stimulating sirtuins (Fig. 1A). It is abundant in berries, grape skins, and red wine^17,18^. Increasing evidence suggests that resveratrol possesses antioxidant and anti-inflammatory properties, along with a range of other potential health benefits^19–23^. However, its bioavailability in the body is relatively low^24^, and its quinone metabolites exhibit cytotoxic effects^25^. It has also been found to be associated with significant side effects in clinical trials^26^. Furthermore, aside from its role in activating SIRT1, resveratrol can also disrupt over a hundred other molecules within cells^27^. The cytotoxicity and multi-target effects of resveratrol limit its use as a specific SIRT1 activator, prompting researchers to explore more effective alternatives for SIRT1 activation.

The first generation of synthetic SIRT1 activators, including SRT1720, SRT2183, and SRT1460 (Fig. 1B), are derived from the imidazo[1,2-b]thiazole core structure. Despite their structural dissimilarity to resveratrol, these compounds effectively activate SIRT1. Nevertheless, further investigations have revealed off-target effects of these activators^28,29^.

The second generation of synthetic SIRT1 activators, represented by SRT2104 and SRT3025, were developed through the optimization of the original imidazo[1,2-b] thiazole scaffold structure (Fig. 1C). Among them, SRT2104 was identified via high-throughput screening of 29,000 compounds. It has demonstrated remarkable stability in human and mouse liver mitochondria and plasma, along with favorable drug exposure profiles. Based on its biochemical activity and pharmacokinetic characteristics, SRT2104 was chosen as a promising drug candidate for further development. Furthermore, in an induced acute inflammation mouse model, SRT2104 exhibited superior in vivo anti-inflammatory activity compared to similar compounds within the similar activation spectrum^19^.

Given the expansive role of SIRT1 in various cellular processes and its potential in disease therapy, and the excellent performance of SRT2104 as the most specific and highly potent agonist of SIRT1, this review aims to assess its therapeutic potential in various diseases, based on a comprehensive analysis of both preclinical and clinical research data. We analyzed the interaction characteristics of SRT2104 and SIRT1, and summarized the molecular mechanisms, preclinical research, and clinical trial advancements of SRT2104 in various diseases by consulting the literature published in databases such as PubMed, Web of Science, and the World Health Organization International Clinical Trials Registry Platform.

## Molecular interaction of SRT2104 with SIRT1

**Figure 2:**
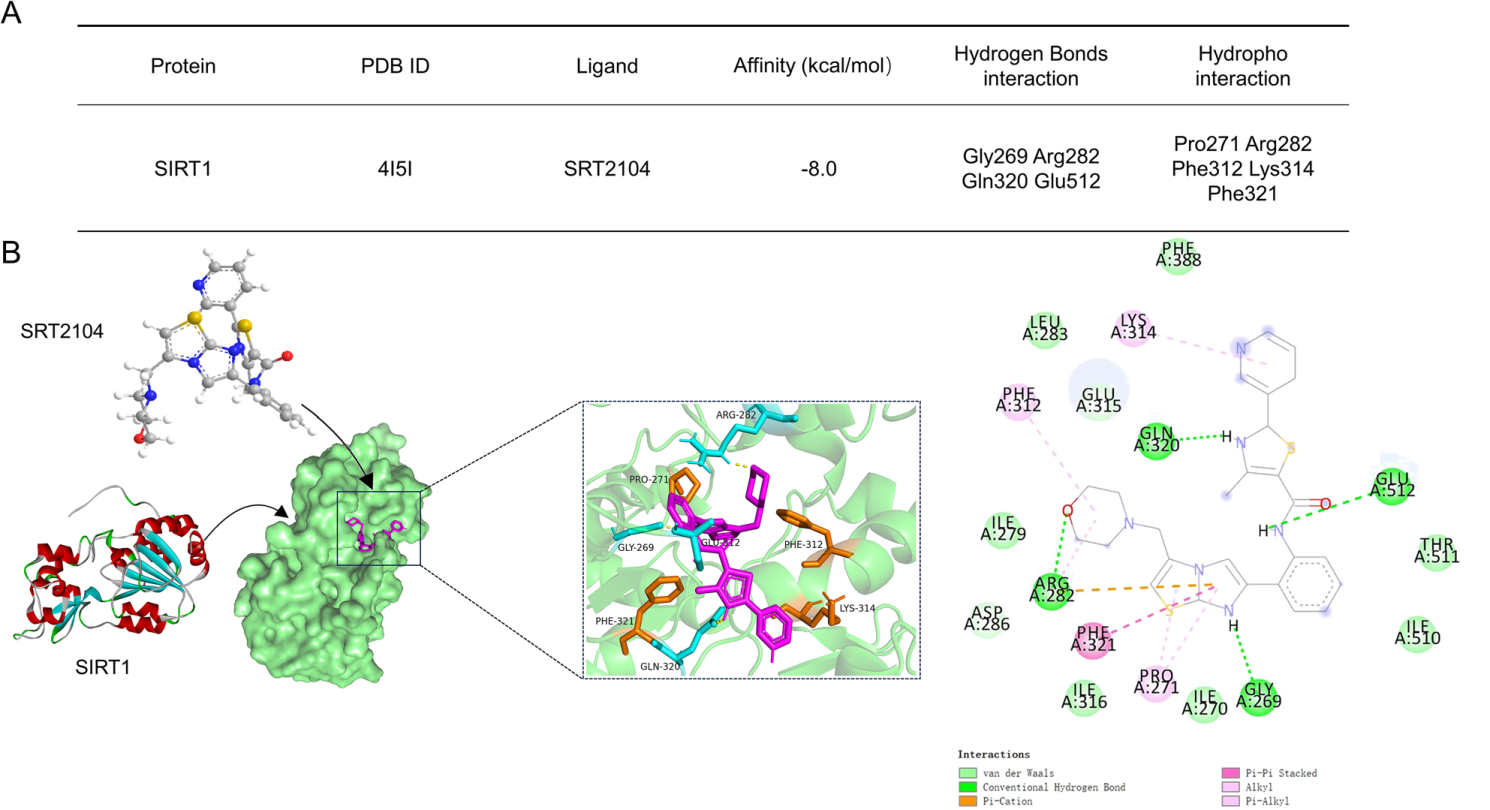
Interaction of SRT2104 with SIRT1. (**A**) Detailed binding profile of SRT2104 to SIRT1. (**B**) Illustrative representation of the molecular interaction between SRT2104 and SIRT1. Molecular docking simulations were conducted utilizing AutoDock software to model the interaction. A grid box measuring 126 Å in each dimension was established, with a grid point spacing of 0.375 Å. During the simulations, the ligands were allowed to flex, whereas the protein's amino acid residues were held fixed. The calculated binding energy served as a measure of affinity. Visualization of the interaction was enhanced using PyMol and Discover Studio, revealing that SRT2104 occupies the hydrophobic pocket of SIRT1. The amine group of SRT2104 forms hydrogen bonds with Gly269, Gln320, and Glu512 in the SIRT1 structure. Additionally, an oxygen atom of SRT2104 engages in a hydrogen bond with ARG282, contributing to the stability of the interaction. Hydrophobic contacts are further augmented by interactions between SRT2104 and SIRT1's Pro271, Arg282, Phe312, Lys314, and Phe321, including Pi-Pi stacking and alkyl interactions. Van der Waals forces involving Ile270, Ile279, Leu283, Ile316, Phe388, Ile510, and Thr511 also play a vital role in sustaining the interaction's stability.

The biological activity of a compound is closely associated with its binding affinity towards the target protein. The stronger the bond formed with the target protein, the lower the binding energy, leading to enhanced molecular stability. To enhance our comprehensive understanding of the interaction between SRT2104 and SIRT1, we conducted detailed molecular docking simulations using the AutoDock software, and employed PyMol and Discover Studio software for visualizing the compound's interactions with key residues. A binding energy below − 4.25 kcal/mol signifies a certain level of binding activity between the ligand and receptor, whereas a binding energy below − 7.25 kcal/mol indicates a robust binding activity^30^. Our findings indicate a robust binding energy of − 8.0 kcal/mol between SRT2104 and SIRT1 illustrating a significant interaction. The comprehensive details regarding the binding information between SRT2104 and SIRT1 were shown in Fig. 2.

## Pharmacokinetics and safety of SRT2104

Compared to the traditional SIRT1 activator resveratrol, SRT2104 has been shown to be 1000 times more effective in activating SIRT1 deacetylase activity, making it a promising molecule for activating SIRT1^31^.

### Clinical Trial 1

To gain a deeper understanding of the pharmacokinetics of SRT2104, GlaxoSmithKline Pharmaceuticals in the United Kingdom conducted a series of Phase I clinical trails^32^. In the dose escalation trial (NCT00933530), healthy subjects were randomly assigned to one of seven dose group (0.03 g, 0.1 g, 0.25 g, 0.5 g, 1.0 g, 2.0 g, and 3.0 g) and orally administered an SRT2104 suspension. Results revealed that peak plasma concentration (Tmax) post single-dose administration ranged from 1 to 3 h, with a half-life (t½) of 8–24 h. Following seven days of consecutive dosing, Tmax was observed between 1 and 2 h, and t½ ranged from 12 to 20 h. Both plasma peak concentration (Cmax) and area under the curve (AUC) increased in a dose-dependent manner with both single and multiple doses. Notably, maximal exposure was reached at the 2.0 g dose without significant differences observed at higher doses. Additionally, subjects demonstrated good drug tolerance over seven days, indicating that a 2.0 g dose is suitable for further clinical research into SRT2104.

### Clinical Trial 2

In an additional clinical investigation (NCT00937872), the primary objective was to ascertain the absolute bioavailability of SRT2104 when administered as a single dose. This study was meticulously designed to provide insights into the pharmacokinetic properties of SRT2104 under controlled conditions. Participants, who were fasting, received an oral dose of 0.25 g SRT2104 suspension, a dosage strategically chosen to balance efficacy and safety.

The outcomes of this trial were pivotal in understanding the pharmacokinetic profile of SRT2104. It was observed that the absolute bioavailability of SRT2104 was approximately 14%, a figure that is critically informative for determining the appropriate dosage for therapeutic efficacy. Furthermore, the study provided valuable data regarding the drug's pharmacokinetics, with an average clearance rate calculated at 404 mL/min. This rate is a crucial parameter in assessing the drug's metabolism and elimination from the body.

### Clinical Trial 3

A clinical study (NCT00938275) investigated the effects of food and gender on SRT2104. The results showed that gender differences had no substantial impact on drug exposure, and there were no substantial differences in the exposure parameters between the suspension and capsule formulations of SRT2104. However, it was observed that taking SRT2104 with a meal increased the exposure parameters (Cmax and AUC) four-fold. Therefore, in future clinical studies, the food effect should be considered to maximize the exposure of SRT2104.

In the aforementioned clinical trials, SRT2104 exhibited favorable tolerability, showing no significant difference in the incidence of adverse reactions compared to the placebo group. Adverse reactions reported among subjects receiving SRT2104 primarily consisted of headaches, gastrointestinal reactions, and nasopharyngitis. Notably, only one case of severe headache was observed, while the remaining adverse reactions were moderate in severity and resolved spontaneously.

### Clinical Trial 4

Another randomized, controlled, double-blind Phase I clinical trial (NCT00964340) was conducted to assess the safety, tolerability, and pharmacokinetic characteristics of SRT2104 in the elderly population^33^. Elderly participants were administered either oral doses of SRT2104 (0.5 g/day or 2.0 g/day) or a placebo that matched the treatment for 28 days. Multiple pharmacodynamic endpoints were assessed through glucose tolerance tests and maximal oxygen consumption tests, with magnetic resonance imaging also used to evaluate visceral and subcutaneous fat. The results demonstrated that SRT2104 could be safely administered to the elderly population with good tolerability. The drug reached peak concentrations within 2–4 h and exhibited a half-life of approximately 15–20 h. Furthermore, it was observed that SRT2104 improved the blood lipid profiles in the elderly participants, leading to reductions in serum cholesterol, low-density lipoprotein (LDL), and triglyceride levels.

These Phase I clinical trials collectively highlight the safety and biological effects of SRT2104 in both healthy and elderly populations. Currently, ongoing clinical trials are being conducted to investigate the potential therapeutic applications of SRT2104 in various disease treatments (Table 1).

**Table 1:** Clinical trials involving SRT2104.

Main ID	Phase of study	Target sample size	Model	Public title	Time	Primary sponsor	Countries of recruitment
NCT01702493	I	16	Healthy male volunteers between 16 and 65 years of age	A Study to Assess the Relative Bioavailability of New Oral Formulations of SRT2104 in Healthy Male Volunteers	2012-10-30 to 2012-12-05	Sirtris, a GSK Company	United States
NCT01453491	I	17	Patients with ulcerative colitis between 16 and 75 years of age	Assess the Safety and Anti-inflammatory Effects of Two Different Doses of SRT2104 in Patients With Ulcerative Colitis	2012-02-13 to 2013-03-18	Sirtris, a GSK Company	United States
NCT01154101	II	40	Moderate to Severe Plaque-Type Psoriasis between 18 and 80 years of age	Study of the Clinical Activity, Safety, and Tolerability of SRT2104 in Subjects With Moderate to Severe Plaque-Type Psoriasis	2010-06-07 to 2011-11-09	Sirtris, a GSK Company	United States
NCT01039909	I	0	Healthy Human Volunteers between 18 and 40 years of age	A Clinical Study to Assess the Effects of SRT2104 Upon Immobilization-Induced Skeletal Muscle Atrophy in Healthy Human Volunteers	2009-12-25	GlaxoSmithKline	–
NCT01031108	I	38	Healthy Cigarette Smokers and Subjects With Type 2 Diabetes Mellitus between 18 and 70 years of age	A Clinical Trial to Assess the Safety of Oral SRT2104 and Its Effects on Vascular Dysfunction in Otherwise Healthy Cigarette Smokers and Subjects With Type 2 Diabetes Mellitus	2010-05-28 to 2011-10-12	Sirtris, a GSK Company	United Kingdom
NCT01018017	II	86	Type 2 Diabetes Mellitus between 18 and 35 years of age	A Clinical Study to Assess the Safety, Tolerability, and Activity of Oral SRT2104 Capsules Administered for 28 Days to Subjects With Type 2 Diabetes Mellitus	2009-12-09 to 2010-05-10	GlaxoSmithKline	Germany
NCT01014117	I	41	Healthy Male Subjects between 18 and 35 years of age	Effect of SRT2104 on Endotoxin-induced Inflammation	2009-12-09 To 2010-05-10	GlaxoSmithKline	Netherlands
NCT00964340	I	24	Healthy Elderly Subjects between 60 and 80 years of age	A Clinical Study to Assess the Safety, Tolerability and Pharmacokinetics of Oral SRT2104 Capsules Administered to Healthy Elderly Subjects for 28 Days	2009-10-01 to 2010-04-27	GlaxoSmithKline	United Kingdom
NCT00937872	I	9	Healthy Male Subjects between 18 and 65 years of age	A Clinical Study to Evaluate the Pharmacokinetics and the Absolute Bioavailability of SRT2104 Given as a 250 mg Oral Suspension and Intravenous Microdose of 100 µg Carbon-14 Radio-labeled SRT2104 in Healthy Male Subjects	2008-11-22 to 2008-12-22	Sirtris, a GSK Company	United Kingdom
NCT00938275	I	20	Normal Healthy Volunteers between 18 and 55 years of age	A Clinical Study to Assess the Effect of Food and Gender on the Pharmacokinetics of SRT2104 Administered as an Oral Suspension or Capsule Formulation to Normal Healthy Volunteers	2009-01-20 to 2009-03-27	Sirtris, a GSK Company	United Kingdom
NCT00937326	II	227	Type 2 Diabetic Human Subjects between 30 to 70 years of age	Clinical Study to Assess the Safety and Pharmacokinetics of SRT2104 in Type 2 Diabetic Human Subjects	2009-08-19 to 2010-09-18	Sirtris, a GSK Company	Bulgaria, Estonia, Hungary, Poland, Romania, Russian Federation, Ukraine, United Kingdom
NCT00933530	I	43	Normal Healthy Male Volunteers between 18 and 55 years of age	A Clinical Study to Assess the Safety and Pharmacokinetics of SRT2104 in Normal Healthy Male Volunteers	2008-05 to 2008-11	Sirtris, a GSK Company	United Kingdom
NCT00933062	I	10	Normal Healthy Male Volunteers between 28 and 60 years of age	Clinical Study to Assess the Pharmacokinetics, Safety and Tolerability of SRT2104 Administered to Normal Healthy Male Volunteers	2009-03-23 to 2009-05-12	GlaxoSmithKline	United Kingdom
NCT00920660	I	20	Healthy Volunteers between 18 and 60 years of age	Clinical Study to Assess the Effects of SRT2104 and Prednisolone on Biomarkers in Blood in Healthy Volunteers	2009-04-06 to 2009-06-12	GlaxoSmithKline	United Kingdom
EUCTR2009-010720-26-GB	II	225	Type 2 Diabetic Human Subjects between 30 and 70 years of age	A Phase II, Randomized, Placebo-Controlled, Double-Blind, Multiple-Dose Clinical Study to Assess the Safety and Pharmacokinetics of SRT2104 in Type 2 Diabetic Human Subjects	2009-08-19 to 2010-09-18	Sirtris Pharmaceuticals, Inc	Bulgaria, Estonia, Hungary, Poland, United Kingdom
EUCTR2009-016537-98-DE	II	80	Type 2 Diabetes Mellitus between 18 and 65 years of age	Study to Assess the Safety, Tolerability and Activity of Oral SRT2104 Capsules Administered for 28 days to Subjects with Type 2 Diabetes Mellitus	2010-03-03 to 2010-12-25	Sirtris Pharmaceuticals Inc	Germany
NTR1971	I	24	Healthy Male Subjects between 18 and 35 years of age	Evaluate single and multiple (seven) oral doses of SRT2104 on the Endotoxin induced inflammatory response in Healthy Male Subjects	2009-11-01 to 2010-01-11	Academic Medical Center, Center of Experimental Molecular Medicine	Amsterdam

## SRT2104 in neurodegenerative diseases

### SRT2104 in Alzheimer's disease

Alzheimer's disease (AD) is a typically neurodegenerative diseases characterized by progressive cognitive impairment and behavioral dysfunction. It mainly affects the elderly and individuals in the preclinical stages of aging. The pathophysiological characteristic of AD encompass the emergence of senile plaques, constituted by extracellular accumulations of β-amyloid (Aβ), as well as the formation of neurofibrillary tangles. These tangles are distinguished by the hyperphosphorylation of Tau proteins, which leads to the entanglement of neuronal fibers and subsequent neuronal attrition within the central nervous system within the central nervous system^34^. Recent investigations have established a correlation between the diminution of SIRT1 expression and the accrual of Aβ in the cerebral cortex of patients afflicted with AD^35^. Consequently, increasing the activity of SIRT1 can potentially exert protective effects against AD^36,37^.

#### Mechanism of action

In a study on Aβ-induced cerebrovascular endothelial injury, SRT2104 dose-dependently enhanced the viability of cerebrovascular endothelial cells, providing significant protection against endothelial cell damage and effectively alleviating the impact of impaired cerebrovascular endothelial cells on memory^38^. These findings suggest that SRT2104, through the regulation of SIRT1, offers a novel therapeutic strategy for AD. However, future clinical trials are warranted to further investigate the safety, efficacy, and mechanisms of action of SRT2104 in AD.

## SRT2104 in Parkinson's disease

Parkinson's disease (PD) represents the second most prevalent neurodegenerative disorder among individuals over the age of 65. The complex molecular mechanisms underlying PD involve oxidative stress, misfolded protein aggregation, and mitochondrial dysfunction^39^. Autophagy-lysosomal dysregulation, a cellular pathway integral to metabolic maintenance and organelle turnover, is intricately associated with these mechanisms. The zinc finger transcription factor ZKSCAN3, characterized by its KRAB and SCAN domains, regulates the transcription of more than 60 genes related to the autophagy-lysosome pathway. Its pivotal role in neuronal survival has been well-documented^40,41^.

### Mechanism of action

1-Methyl-4-phen-1,2,3,6-tetrahydropyridine (MPTP) and its active metabolite 1-methyl-4-phenylpyridinium ion (MPP+) are often used in inducing mammalian models^42^. In an in vitro model of PD induced by MPTP/MPP+, it was observed that the autophagy level was inhibited, and ZKSCAN3 accumulated in the cell nucleus. However, SRT2104 promoted the removal of ZKSCAN3 from the cell nucleus, restored the level of cellular autophagy, alleviated autophagic lysosomal damage, and protected dopamine neurons against MPP+-induced oxidative stress.

### Preclinical study

Furthermore, in an MPTP-induced PD mouse model, SRT2104 promoted the restoration of autophagy levels in the midbrain ventral lateral area, reduced the death of dopamine neurons, and mitigated impairments in coordination and motor abilities in PD mice^43^. These studies substantiate that SRT2104 has the potential to alleviate neurofunctional impairments in PD by restoring autophagic dysfunction, suggesting its potential as a therapeutic drug in Parkinson's disease.

## SRT2104 in brain ischemia/reperfusion injury

Brain ischemia/reperfusion injury, a prevalent global neurological disorder, interrupts cerebral blood flow. This disruption leads to deficits in oxygen and nutrient delivery essential for cellular function maintenance^44^. As the major line of defense in the central nervous system, microglial cells display heterogeneity under microenvironmental disturbances and can differentiate into M1-type pro-inflammatory or M2-type anti-inflammatory phenotypes. M1 microglia secrete pro-inflammatory cytokines, resulting in brain damage, while M2 microglia secrete anti-inflammatory cytokines and neurotrophic factors, promoting brain repair^45^. Therefore, one potential approach for treating brain ischemia/reperfusion injury is to modify the polarization of microglia from the M1 phenotype to the M2 phenotype^46^.

### Mechanism of action

Fu et al. conducted a study that investigated the neuroprotective effects of SRT2104 in an oxygen–glucose deprivation/reperfusion (OGD/R) induced cellular injury model. The study demonstrated that SRT2104, through its stimulation of SIRT1 expression, hampers the activation of the NF-κB pathway and effectively modifies microglial polarization from the detrimental M1 phenotype to the beneficial M2 phenotype. Furthermore, this modulation of microglial polarization directly and indirectly prevents neuronal and microglial cell death caused by OGD/R^44^.

These findings suggest that SRT2104 exerts neuroprotective effects by modifying microglial polarization, highlighting its potential as a therapeutic agent for brain ischemia/reperfusion injury.

## SRT2104 in Huntington's disease

Huntington's disease (HD) is a neurodegenerative disorder caused by the extension of polyglutamine sequence in the N-terminal of huntington protein. The clinical characterizations of HD primarily manifest as motor disturbances, cognitive impairments, and psychiatric disorders. Currently, there are no pharmacological agents officially sanctioned for the therapeutic management of HD^47^. Studies have shown that SIRT1 overexpression improves survival and exhibits neuroprotective effects in HD mouse models, while the absence of SIRT1 exacerbates disease progression^48,49^.

### Preclinical study

In 2014, Jiang et al. in a pivotal study conducted in 2014, Jiang et al. provided empirical evidence demonstrating that SRT2104 successfully traversed the blood–brain barrier. Subsequent observations revealed that this penetration was associated with a notable reduction in cerebral atrophy. Furthermore, the treatment led to enhancements in motor function proficiency, which was a significant finding considering the debilitating motor deficits characteristic of Huntington's disease. Most importantly, the administration of SRT2104 was correlated with a prolonged lifespan, thereby highlighting its potential therapeutic efficacy^50^. Although further investigations are needed to elucidate the underlying molecular mechanisms through which SIRT1 regulates HD in mice, the positive effects observed with SRT2104 provide compelling evidence for the potential of SIRT1 activation as a novel therapeutic strategy for the management of HD.

## SRT2104 in optic nerve degenerative diseases

Glaucoma is an optic nerve degenerative disease induced by various risk factors such as elevated intraocular pressure. The underlying pathology involves the progressive loss of retinal ganglion cells and axons. In the realm of therapeutic strategies, neuroprotection, which focuses on preventing neuronal death, has proven to be an effective treatment strategy for glaucoma^51–53^. In this context, SRT2104 has garnered attention due to its neuroprotective properties, which have shown efficacy in a spectrum of central nervous system disorders, including AD, PD, and HD. This raises the intriguing possibility of repurposing SRT2104 for use in glaucoma and similar optic nerve degenerative diseases.

### Mechanism of action

Recently, Bai et al. conducted in-depth research on the protective effects of SRT2104 on the retina under ischemia/reperfusion (I/R) injury and its underlying mechanisms. They observed a significant decrease in the expression of SIRT1 protein following I/R-induced injury. Administration of SRT2104 effectively enhanced the expression of SIRT1 protein, exerting substantial protective effects on both the retina and neurons. It partially restored retinal function after I/R injury and significantly alleviated cell apoptosis and aging induced by I/R. Moreover, SRT2104 demonstrated a remarkable reduced neuroinflammation after I/R injury. Mechanistic studies further revealed that SRT2104 could effectively reverse the acetylation of p53, NF-κB p65, and Signal Transducer and Activator of Transcription 3 (STAT3) induced by I/R^54^.

### Preclinical study

In a comprehensive preclinical investigation, the efficacy of SRT2104 was evaluated in a rat model subjected to optic nerve degeneration induced by tumor necrosis factor (TNF). The study revealed that SRT2104 exerted a dose-dependent protective effect, ameliorating the damage to the optic nerve typically provoked by TNF. This salutary action was closely associated with the enhancement of autophagic processes within the optic nerve tissues. Detailed molecular analyses suggested that the upregulation of autophagy, which is crucial for cellular homeostasis and the removal of damaged cellular components, was directly mediated by the administration of SRT2104^55^.

Taken together, these findings suggest that SRT2104 exerts neuroprotection in the nerve by enhancing SIRT1-mediated deacetylation, inhibiting neuronal apoptosis and aging, reducing neuroinflammation, and promoting autophagy in the optic nerve.

## SRT2104 in depression

Depression, a chronic mental health disorder, is frequently marked by a constellation of symptoms, notably including impaired cognitive functioning and altered thought processes^56^. Individuals with depression exhibit a decrease in hippocampal volume, reduced neuronal density, and impaired complexity of dendritic spines^57^.

In 2015, the China-Oxford-Virginia Commonwealth University Experimental Gene Epidemiology Research team conducted a DNA sequence analysis of 5303 Chinese women with severe depression and 5337 control participants. The results revealed a significant correlation between the SIRT1 gene and the susceptibility to developing depression^58^. Furthermore, in 2016, Luo et al. investigated the blood samples of 50 patients with severe depression and found a 37% decrease in the expression of the SIRT1 gene compared to the control group^59^. Collectively, these clinical research findings suggest a strong relationship between the onset of depression and the expression of the SIRT1 gene.

### Preclinical Study 1

Animal models have further supported these findings by showing that mice subjected to chronic unpredictable mild stress (CUMS) display depressive symptoms, cognitive impairments, and memory deficits. Moreover, the expression SIRT1 was found to be downregulated.

However, intervention with SRT2104 has shown promising results in alleviating depressive-like behaviors in mice. In particular, it significantly prolongs the struggling time in the forced swimming test and tail suspension test, suggesting a considerable improvement in depressive-like behavior. Additionally, SRT2104 effectively ameliorates dendritic atrophy in the hippocampus of depressed mice^60^.

### Preclinical Study 2

Similar alterations in dendritic morphology have been observed in a rat model of diabetes-induced cognitive impairment. By administering SRT2104 via intracerebroventricular injection to rats with cognitive impairment, researchers have found that it promotes the growth of dendrites and increases density in the hippocampus, which leads to improvements in cognitive function^61^.

On the whole, these studies propose that a deficiency in SIRT1 expression within the hippocampal region can influence cognitive function and dendritic structure. Conversely, the administration of SRT2104 appears to have a positive impact on depressive-like behaviors and regulates dendritic structure in the hippocampal.

### Mechanism of Action 1

The medial prefrontal cortex (mPFC) is a crucial neural center involved in the regulation of cognition and behavior, playing a pivotal role in the pathogenesis of depression^62^. Knockdown of SIRT1 in the mPFC of adult mice induces depressive behavior. Conversely, administration of SRT2104 into the mPFC or lateral ventricle of wild-type mice effectively ameliorates depression-like behaviors, such as anhedonia and behavioral despair triggered by CUMS. Moreover, SRT2104 treatment normalizes excitability and synaptic transmission in mPFC pyramidal neurons^63^. These findings suggest that SRT2104 may exert antidepressant effects by modulating excitability and synaptic transmission in mPFC pyramidal neurons through the activation of SIRT1.

### Mechanism of Action 2

In the brain, this inflammatory response is closely linked to the polarization state of microglial cells. Activated M1 microglia contribute to inflammation, while M2 microglia effectively suppresses the inflammatory response^64^. The transition of microglial cells in the hippocampus of mice to an M2 phenotype effectively alleviates depressive-like behaviors in mouse models^65^. Elevated levels of pro-inflammatory cytokines in the blood of patients with depression were also observed^66,67^.

SRT2104 significantly reduces the levels of pro-inflammatory cytokines, such as Interleukin-6 (IL-6), Interleukin-1 Beta (IL-1β), and Inducible Nitric Oxide Synthase (iNOS), while increasing the levels of anti-inflammatory cytokines, including Interleukin-10 (IL-10), Transforming Growth Factor Beta (TGF-β), and Arignase1. Moreover, treatment with SRT2104 leads to decreased expression of the M1 marker Major Histocompatibility Complex Class II (MHC-II) and increased expression of the M2 marker Cluster of Differentiation 206 (CD206) in the hippocampus of depressed mice, indicating that SRT2104 promotes the transition of M1 microglia to an M2 phenotype. This therapeutic effect is mediated by the Glycogen Synthase Kinase 3 Beta-Phosphatase and Tensin Homolog (GSK3β-PTEN) signaling pathway^68^.

### Preclinical Study 3

Postpartum depression (PPD) is a depressive disorder occurring post-childbirth, characterized by substantial depressive symptoms. Research indicates a notable reduction in glucocorticoid receptor (GR) expression in depression animal models' brain tissues^69,70^. Heterozygous GR knockout mice display depressive-like behavior^71^, underscoring GR's significant role in depression's development. Additionally, GR's physical interaction with SIRT1 enhances GR transcription activity has been evidenced^72^.

Wang et al. induced PPD in rats through estrogen withdrawal and observed a significant decrease in GR expression. However, the injection of SRT2104 into the hippocampus of rat models suffering from PPD resulted in the successfully activated SIRT1, leading to targeted the regulation of GR. Consequently, the increased expression and activity of GR were accompanied by noteworthy behavioral changes in PPD. These findings indicate a potential involvement of the SIRT1-GR signaling pathway in the inhibition of PPD. Therefore, SRT2104 may serve as a prospective therapeutic strategy for PPD^73^.

## SRT2104 in diabetic complications

Diabetic vascular complications, a prevalent chronic condition induced by diabetes, pose a significant threat to individuals with diabetes. Research indicates that diabetic patients have a 50–80% probability of experiencing cardiovascular complications, with about 70% of these patients die due to these complications^74^. In this context, it is crucial to explore potential therapeutic interventions that can mitigate the progression of diabetic vascular complications. High blood glucose found to impede the expression of SIRT1 and induce cellular senescence in vascular cells, thereby exacerbating the development of diabetic vascular complications. Consequently, it is hypothesized that the activation of SIRT1 could yield outcomes in managing these complications^75–77^.

### Preclinical Study 1

In C57BL/6 mice, diabetes induced by streptozotocin led to increased aortic contractility, oxidative stress, inflammation, P53 hyperacetylation, and reduced SIRT1 protein. A 24 weeks treatment with 100 mg/kg of SRT2104 resulted in a 3.79-fold increase in SIRT1 protein levels in the aorta of diabetic mice. Notably, this treatment significantly alleviated symptoms associated with endothelial dysfunction in the aforementioned diabetic mice, including increased arterial contraction, oxidative stress, and inflammation^78^. Consequently, SRT2104 demonstrated pronounced efficacy in mitigating endothelial dysfunction in the aorta, underscoring its therapeutic potential in diabetic models.

### Mechanism of Action 1

The team further explored the underlying mechanisms. In endothelial cells (ECs) treated with high glucose (HG), both P53 siRNA and SRT2104 upregulated SIRT1 and inhibited P53 acetylation, oxidative stress, and inflammation. However, SRT2104 did not enhance these effects in the presence of P53 siRNA. P53 activation with nutlin3a negated SRT2104's protective effects against HG-induced stress and inflammation and increased aortic contractility and endothelial stress in healthy mice^78^. This study suggests P53 deacetylation as a key mechanism in SRT2104's protection against diabetic aortic endothelial dysfunction and underscores P53's pathogenic role in this context.

### Clinical Trial 1, 2

However, two clinical trials assessing the effects of SRT2104 on endothelial function in patients with type 2 diabetes and healthy smokers failed to produce significant results^79,80^. It is important to acknowledge that the duration of SRT2104 treatment in the two trials was restricted to only 28 days. This limited timeframe may have impeded the detection of potential protective effects on endothelial function. Currently, there is a lack of consensus regarding the optimal treatment duration for SRT2104 in clinical practice. In light of this, the effects of SRT2104 on endothelial function should be further investigated through in a larger-scale and longer-term clinical trials that include appropriate treatment duration and follow-up protocols.

### Preclinical Study 2

Prolonged treatment with SRT2104 (24 weeks) in diabetic animal models exhibited significant improvements in diabetes-related endothelial dysfunction in the aorta^78^, testicular cell death^81^, and kidney injury^82^. These studies suggest that long-term treatment with SRT2104 may lead to more favorable therapeutic outcomes. However, early intervention is also crucial for achieving better treatment results besides extending the treatment duration. This is because once the typical pathological features of diabetes are established, the condition becomes nearly irreversible. Therefore, long-term preventive treatment with SRT2104 is of utmost importance in diabetes. Furthermore, when considering the future of SRT2104 in clinical trials for other chronic conditions, the consideration of treatment duration may also be essential.

### Mechanism of Action 2

Further investigations into the impact of SRT2104 on diabetic nephropathy and the associated signaling pathways involved were conducted. In wild-type mice, SRT2104 was found to enhance renal SIRT1 activity and deacetylated p53, thereby activating the Nuclear Factor Erythroid 2-Related Factor 2 (Nrf2) antioxidant signaling pathway. This pathway, which plays a critical role in regulating oxidative stress, offered significant protection against various diabetes-induced renal complications, including oxidative stress, inflammation, fibrosis, glomerular remodeling, and proteinuria. These findings confirm the beneficial effects of SRT2104 on diabetic nephropathy and shed light on the mechanisms underlying the modulation of the SIRT1/p53/Nrf2 pathway^82^.

### Preclinical Study 3

Diabetes has been found to have multifaceted effects on male reproductive function. Apart from causing erectile dysfunction, long-term diabetes can induce testicular cell apoptosis, leading to infertility^83^. Diabetic male mice exhibited significant oxidative stress, endoplasmic reticulum stress, and apoptosis in testicular cells. By promoting the expression of the SIRT1 protein, SRT2104 mitigated diabetes-induced oxidative stress and endoplasmic reticulum stress in the testes, exerting a remarkable protective effect on testicular cells^81^. The administration of SRT2104 presents a potential new therapeutic strategy for addressing diabetes-induced male infertility.

### Preclinical Study 4

SRT2104 has been mentioned to have a significant improvement effect on diabetic peripheral neuropathy, as mentioned in the previous text. However, SRT2104 can also produce beneficial effects in diabetic central neuropathy.

In a diabetic rat model induced by streptozotocin, cognitive dysfunction was observed after 8 weeks, characterized by reduced dendritic branching and lower dendritic spine density in the hippocampus, indicative of cognitive impairment^61^. Notably, the administration of SRT2104 into the lateral ventricle resulted in an increase in hippocampal dendritic length and dendritic spine density. This improvement was correlated with the activation SIRT1 by SRT2104, ultimately leading to enhanced cognitive function. Mechanistic studies investigations have proposed that SIRT1 influences hippocampal dendritic morphology through the regulation of the Target of Rapamycin Complex 1/Cyclic AMP-Responsive Element-Binding Protein (TORC1/CREB) signaling pathway. Hence, SRT2104 may beneficially influence central neuropathy in diabetes by ameliorating hippocampal dendritic alterations and enhancing cognitive function, mediated by SIRT1 activation and modulation of related signaling pathways.

### Clinical Trial 3

A randomized, controlled trail (NCT00937326) involving patients with type 2 diabetes investigated the effects of SRT2104 administration. Over a 28-day period, participants received varying doses of SRT2104, ranging from 0.25 to 2.0 g/day. The results indicated a positive impact on blood lipid profiles, such as reductions in LDL cholesterol and triglycerides. However, the treatment did not significantly alter blood glucose levels or insulin sensitivity in these patients, suggesting limited efficacy in glycemic control^84^.

### Clinical Trial 4

In a subsequent clinical trial (NCT01031108), researchers assessed the impact of SRT2104 on cardiac metabolism in individuals with type 2 diabetes. Participants were administered a short-term treatment of 2.0 g/day of SRT2104. While the compound was well-tolerated, with minimal adverse effects reported, it failed to produce significant improvements in cardiovascular health markers. This included parameters such as cardiac output, myocardial energy expenditure, and overall cardiovascular efficiency^79^.

### Clinical Trial 5

Another arm of the study (NCT01031108) focused on the potential vascular benefits of SRT2104, particularly its effect on arterial stiffness, a common complication in type 2 diabetes. Participants undergoing short-term treatment with SRT2104 exhibited a noticeable improvement in arterial stiffness measures. This outcome was quantified using established clinical indicators, such as pulse wave velocity and augmentation index, which reflect arterial health and flexibility^85^.

## SRT2104 in lipid metabolism and cardiovascular disease

In clinical practice, arterial stiffness is increasingly recognized as a significant indicator of cardiovascular risk. Smoking and diabetes are prominently acknowledged as major contributors to the development of cardiovascular disease. Current literature robustly establishes the association between smoking, diabetes, arterial stiffness, and endothelial dysfunction, highlighting their intertwined roles in cardiovascular pathology^86–89^.

### Clinical Trial 1

A clinical trial (NCT01031108) at the Cardiovascular Science Centre of the University of Edinburgh focused on the impact of oral administered SRT2104 on cardiovascular function in healthy smokers. This double-blind, randomized trail involved administering 2.0 g of SRT2104 or a matched placebo daily for 28 days. All participants demonstrated tolerability throughout the intervention, with no significant adverse events reported^80^.

In this trail, the SRT2104 group exhibited significant improvements in lipid profiles compared to the placebo group, featuring an average reduction of 11% in low-density lipoprotein and a 7% decrease in total cholesterol levels^80^. A separate trial involving in elderly individuals also observed lipid profile improvements with SRT2104^33^. Although the exact mechanism behind these lipid reductions is unclear, animal models suggest that SIRT1 activation can decrease triglyceride levels and enhance insulin sensitivity^90^. SRT2104 may positively regulate liver X receptors (LXRs), key players in lipid metabolism and cholesterol efflux. Li et al.'s study supports this by showing SIRT1 deacetylation of LXRs affecting cholesterol transport and metabolism^91^.

Despite these findings, the trial did not yield significant improvements in vascular reactivity, endothelial function, or platelet activation assessments^80^. This could be due to the limited duration of SRT2104 exposure, suggesting a need for further investigation into the drug's pharmacokinetics and pharmacodynamics. A longer treatment period might be necessary to observe effects on blood vessels and endothelial cells. The study's small sample size might also have influenced these results.

In conclusion, the trial confirmed the safety and tolerability of 2.0 g/day oral SRT2104 in healthy smokers, with a positive impact on blood lipid profiles. However, its effects on vascular, endothelial, or platelet function compared to placebo remain unproven, highlighting the need for further research.

### Clinical Trial 2

In a recent clinical trial (NCT01031108), the effects of oral SRT2104 on arterial compliance were investigated in both smokers and type 2 diabetes patients. The study involved 39 participants, comprising 24 healthy smokers and 15 type 2 diabetes patients. They were randomly assigned to either a treatment or placebo group in a double-blind, placebo-controlled design. The treatment group received oral SRT2104 at a dosage of 2.0 g/day for 28 days, while the placebo group received a matching placebo.

Results show that SRT2104 significantly reduced arterial stiffness in both healthy smokers and type 2 diabetes patients. However, no significant difference was observed in pulse wave velocity when compared to the placebo group^85^.

These findings, derived from a short treatment period and a small sample size, should be interpreted with caution. Further research is necessary to delineate the mechanism through which SRT2104 improves arterial compliance and to assess its long-term vascular health effects. Further studies, particularly those larger cohorts and extended treatment durations, are required to fully understand SRT2104’s therapeutic potential in improving arterial compliance in smokers and type 2 diabetes patients.

## SRT2104 in musculoskeletal diseases

The maintenance of bone homeostasis relies on the balance between osteoblast-mediated bone formation and osteoclast-mediated bone resorption. The dysregulation of osteoblast and osteoclast activity leads to abnormal bone remodeling, which is a common mechanism underlying osteoporosis^92^. The crucial role of SIRT1 in maintaining bone homeostasis has been demonstrated through various studies. For instance, the deletion of the SIRT1 gene in young canines resulted in delayed trabecular and cortical bone development^93^. SIRT1-deficient mice exhibit decreased bone mass and an imbalance between osteoblasts and osteoclasts, providing additional evidence for the importance of SIRT1 in bone health^94^.

### Preclinical Study 1,2

SRT2104 has exhibited encouraging outcomes in enhancing bone health. It was found to inhibit inflammatory responses and extend the lifespan of mice on a standard diet. This extension in lifespan was accompanied by improvement in motor coordination, endurance, and bone density. Moreover, SRT2104 effectively suppressed osteoclastogenesis in a model of muscle atrophy^90^. Notably, it also inhibited osteoclast formation trigered by the receptor activator of nuclear factor-kappa B ligand (RANKL) in bone marrow macrophages, highlighting its potential role in bone remodeling and maintenance^95^.

### Mechanism of Action 1

However, recent research by Zhang et al. raised concerns regarding the targeted distribution of SRT2104 within bone tissue when administered orally or intravenously. To address this, they developed a novel delivery system utilizing mineral-coated decellularized matrix particle for local sustained release SRT2104. This system exhibited near zero-order release kinetics and significantly promoted osteogenic differentiation and mineralization of bone marrow mesenchymal stem cells. It also inhibited osteoclast function and excessive generation by deacetylating Forkhead Box O1 (FoxO1) and Nuclear Factor of Activated T-cells, cytoplasmic 1 (NFATc1) expression^96^.

### Mechanism of Action 2

In the context of bone marrow mesenchymal stem cell differentiation, both osteogenic and angiogenic, as induced by bone morphogenetic protein 9 (BMP9), SRT2104 has been found to augment the expression of key osteogenic markers. these include Runt-related transcription factor 2 (RUNX2), Collagen, Type I Alpha 1 (COL1A1), Osteocalcin (OCN). Concurrently, it also elevates the levels of proteins associated with bone vascularization, namely cluster of differentiation 31 (CD31) and vascular endothelial growth factor A (VEGFA). This enhancement in expression facilitates BMP9-induced osteogenic and angiogenic differentiation, primarily through the activation of the BMP/Smad and BMP/MAPK pathways^97^.

These findings underscore SRT2104’s ability to boost osteoblastic activity and impede osteoclast activation and proliferation, making it as a promising candidate for the treatment of osteoporosis. Zhang et al.'s targeted delivery system further enhances SRT2104's efficacy in bone health. Further studies and clinical trials are needed to confirm SRT2104's effectiveness and safety in osteoporosis treatment.

### Mechanism of Action 3

SRT2104’s therapeutic effects on osteoarthritis (OA) may be attributed to multiple mechanisms, including the reduction of cartilage-degrading enzymes, apoptotic markers, acetylated NF-κB p65, and inflammation factors (IL-1β and IL-6). Additionally, SRT2104 has been shown to promote the conversion of M1 to M2 macrophages in the synovium, which contributes to alleviation of OA. Remarkably, both intraperitoneal and intra-articular administration of SRT2104 have exhibited efficacy in alleviating mouse osteoarthritis study. Therefore, these findings suggest that SRT2104 holds promise as a potential therapeutic agent for the treatment of osteoarthritis^98^.

### Preclinical Study 3

In a microgravity, the unloading of the astronauts’ hind limbs induces skeletal muscle atrophy. This phenomenon is primarily attributed to the detrimental effects of the microgravity environment on mitochondrial in skeletal muscles, leading to an imbalance between oxidative stress and antioxidant defense^99^. Consequently, restoring mitochondrial function and preventing reactive oxygen species generation are important in alleviating skeletal muscle atrophy during spaceflight. Furthermore, the activation of SIRT1, a protein involved in skeletal muscle repair, has shown promising effects on mitigating skeletal muscle impairment^100^.

Recently investigations have focused on evaluating the impact of SRT2104 on skeletal muscle mitochondrial function. The findings revealed that in the unloaded gastrocnemius muscle of rats, administration of SRT2104 significantly improved mitochondrial efficiency by reducing proton leak and promoting oxygen flux. Confirmed by the notable enhancement in cytochrome c oxidase activity, these results indicate that SRT2104 intervention facilitates enhanced mitochondrial metabolism within the gastrocnemius muscle, consequently boosting energy production. Additionally, presence of SRT2104 increased the activity of superoxide dismutase, a key enzyme responsible for neutralizing oxidative factors, suggesting an enhanced ability to counteract oxidative stress within the in the gastrocnemius muscle. Most importantly, the administration of SRT2104 effectively ameliorated skeletal muscle atrophy induced by unloading. The findings collectively demonstrate that SRT2104 exhibits a substantial ability to alleviate skeletal muscle atrophy by enhancing mitochondrial function and reducing oxidative stress^101^.

## SRT2104 in chronic obstructive pulmonary disease

Chronic obstructive pulmonary disease (COPD) is characterized by irreversible airflow obstruction and progressive decline lung function. The primary cause of long-term airflow limitation is the destruction of alveolar structures, resulting in enlargement of airspace and degradation of lung elastic tissue^102^. In mammals, the alveolar epithelial cells consist of type I (AECI) and type II alveolar epithelial cells (AECII). AECII play crucial roles in the secretion of pulmonary surfactants, maintenance of alveolar homeostasis, gas exchange, and enhancement of lung tissue repair capacity^103^. Emerging evidence suggests that SIRT1 is involved in the progression of COPD through various mechanisms and cellular pathways. Reduced levels of SIRT1 have been found to promote aging and functional abnormalities of AECII in COPD patients^104,105^.

### Preclinical study

In a rat model of emphysema induced by cigarette smoke extract (CSE) and intratracheal lipopolysaccharide (LPS) instillation, the expression levels of Surfactant Protein A (SPA) and Surfactant Protein C (SPC) in lung tissues were found to be reduced, indicating damage to AECII. However, treatment with SRT2104 significantly alleviated the pathological features of emphysema and improved lung function parameters, including airway resistance, lung dynamic compliance, and peak expiratory flow rate. Moreover, SRT2104 upregulated the expression SPA, SPC, SIRT1, and FoxO3a (a non-histone substrate of SIRT1), reduced the activity of senescence-associated β-galactosidase (SA-β-gal, a key biomarker of cellular senescence), and increased the level of p53 deacetylation, another non-histone substrate of SIRT1^106^. These results indicate a significant reduction in senescent alveolar cells in lung tissues after SRT2104 treatment.

### Mechanism of action

Yuan et al. isolated primary AECII from lung surgery patients and induced aging in vitro with cigarette smoke extract (CSE). They observed diminished SIRT1 binding to the FoxO3a promoter and increased binding to p53 in aged AECII. Treatment with SRT2104 significantly enhanced SIRT1’s association with the FoxO3a promoter, suggesting SIRT1’s role in modulating transcription complexes at FoxO3a and p53 promoters in AECII. Stimulation with CSE upregulated SIRT1-p53 promoter interaction, while SRT2104 did the same for SIRT1- FoxO3a^107^. Given the reduced expression of SIRT1 and FoxO3a in COPD patients' lungs, which correlates with compromised AECII function, attenuating AECII aging could improve lung repair and function^105,108^.

In summary, these findings indicate that SRT2104 may counteract AECII aging or enhance their repair via the SIRT1/FoxO3a and SIRT1/p53 pathways, offering a potential avenue to slow COPD progression and a new therapeutic strategy for its management.

## SRT2104 in sepsis

Sepsis, a refractory disease resulting from severe infection, is characterized by high incidence and mortality rates. It manifests as a potentially life-threatening systemic inflammatory response, also known as systemic inflammatory response syndrome. The expression level of SIRT1 varies based on the immune status. Extensive in vitro and in vivo studies have shown that levels of SIRT1 decrease during acute inflammatory reactions^109–112^, whereas upregulation of SIRT1 demonstrates robust anti-inflammatory activity^113–116^. As such, SIRT1 is regarded as a molecular switch that regulates inflammatory responses.

### Clinical trial

A randomized, double-blind, placebo-controlled trail (NCT01014117) investigated the anti-inflammatory properties of SRT2104 in LPS induced sepsis in human.. The outcomes revealed that SRT2104 significantly reduced the LPS-induced secretion of IL-6 and IL-8 and lowered C-reactive protein levels compared to placebo. However, it did not affect IL-10 and TNF-α release. SRT2104 also attenuated the coagulation response to LPS, though white blood cell counts remained unchanged between the SRT2104 and placebo groups^117^.

The anti-inflammatory effects of SRT2104, documented in both mice^90^ and humans^117^, might involve modulating tissue macrophages and other extravascular leukocytes to curb the release of cytokines triggered by intracellular LPS. One hypothesized pathway is SIRT1's deacetylation of the RelA/p65 NF-κB subunit, inhibiting NF-κB transcriptional activity and thus diminishing IL-6 production^7^.

Moreover, recent insights suggest SRT2104's anti-inflammatory effects may be partially attributed to modulating pyruvate kinase M2 (PKM2), a key inflammatory regulator in conditions such as sepsis, asthma, and encephalomyelitis. Elevated PKM2 levels during inflammation can be counteracted by autophagy^118–120^, highlighting another potential mechanism for SRT2104's therapeutic action.

### Mechanism of action

In the LPS-induced murine model of sepsis, a study noted an upregulation of Pyruvate Kinase M2 (PKM2) and a concomitant downregulation of the autophagy marker LC3B-II. Upon SRT2104 administration, these changes were significantly attenuated. This therapeutic intervention led to reduced inflammatory responses, ameliorated lung injury, and a marked improvement in mouse survival rates.

To ascertain whether SRT2104’s inhibition of PKM2 was autophagy-dependent, Zhao et al. implemented concurrent treatment with the autophagy inhibitor 3-methyladenine (3-MA) and SRT2104. Their data suggested that autophagy inhibition by 3-MA could reverse SRT2104-induced downregulation of PKM2 and the mitigation of inflammatory responses. These findings suggest a mechanism by which SRT2104 modulates PKM2 and autophagy to exert anti-inflammatory effects^121^.

## SRT2104 in ulcerative colitis

Ulcerative colitis is a chronic condition characterized damage to the mucosa and recurrent inflammation of gastrointestinal tract in the colon.

### Clinical trial

A phase I clinical trial (NCT01453491) assessed the safety, tolerability, and therapeutic effects of SRT2104 in patients with mild to moderate ulcerative colitis. In this trail, 31 patients were randomized in a 1:1 ratio to receive oral doses of SRT2104 at dosages of 50 mg/day or 500 mg/day over a span of 8 weeks. The results showed that SRT2104 was generally well-tolerated, with drug exposure increasing in a dose-dependent manner. Notably, colonic exposure to the drug was substantially higher than plasma exposure, ranging from 140 to 160-fold.

Despite this, the trail did not yield significant clinical efficacy in improving ulcerative colitis symptoms, as assessed by endoscopic examination. Interestingly, a reduction in fecal calprotectin levels, a marker of colitis disease activity, was observed post-treatment with SRT2104, with levels decreasing by approximately 75%. However, these levels remained significantly elevated, approximately four-fold higher than the normal range, even after the treatment period^122^.

It is important to consider the limitations inherent in this study, such as the small cohort size and the absence of a placebo control group. These factors necessitate a cautious approach when considering the reliability and applicability of the results. Further research, ideally with larger sample sizes and a placebo-controlled design, is needed to substantiate these findings and fully ascertain the therapeutic potential of SRT2104 in ulcerative colitis.

## SRT2104 in psoriasis

Psoriasis, a papulosquamous skin disease initially attributed to abnormalities in the keratinocyte differentiation, is now acknowledged as the most prevalent chronic immune-mediated inflammatory skin disorder^123,124^. For patients with moderate to severe psoriasis who do not respond adequately or have contraindications or to phototherapy and conventional systemic therapies, biologics are typically considered. Commonly used biologics include tumor necrosis factor alpha (TNF-α) inhibitors (e.g., Adalimumab) and Interleukin-17 (IL-17) inhibitors (e.g., Secukinumab)^125,126^. Notably, both TNF-α and IL-17 have been found to be regulated by SIRT1, suggesting that targeting SIRT1 may have the potential to block the inflammatory response at an upstream level^127–129^.

### Clinical trial

A randomized, double-blind, placebo-controlled phase II clinical trial (NCT01154101) investigating the efficacy of SRT2104 in moderate to severe psoriasis has been completed^130^. The study enrolled forty patients with a history of plaque psoriasis for at least 6 months. Eligible patients, with a Psoriasis Area and Severity Index (PASI) score of 10 or higher, were randomized in a 4:1 ratio to receive either oral SRT2104 at doses of 250 mg/day, 500 mg/day, 1000 mg/day or a placebo for 84 days. The average PASI scores in the placebo group was 15.65, while the average PASI scores in the 250 mg, 500 mg, and 1000 mg groups were 14.41, 13.32, and 11.43, respectively.

Skin biopsy analysis revealed significant improvement in 35% of patients receiving the SRT2104 treatment. Transcriptomic analysis further demonstrated that SRT2104 reduced the expression of TNF-α and IL-17 genes while suppressing the expression of epidermal keratinocyte-related genes, including Small Proline-Rich Protein 2c (SPRR2c), Serpin Family B Member 4 (SERPINB4), and Serpin Family B Member 3 (SERPINB3). Adverse reaction were reported in 27 participants, including those in the placebo group (69% in total). Most adverse were mild to moderate, with the most common being dizziness (8%), headache (8%), upper respiratory tract infection (8%), and psoriatic arthritis (8%).

The improvement in clinical scores of psoriasis observed with SRT2104 may be attributed to its combined effects of inhibiting the TNF-α and IL-17 inflammatory signaling pathways and regulating epidermal keratinocyte function. Considering the significant adverse reactions associated with approved treatments for moderate to severe psoriasis, as well as the evident clinical activity and good tolerability and safety profile of SRT2104, further research exploring its use in the treatment of psoriasis is warranted.

## SRT2104 in female reproductive system diseases

Preeclampsia, a distinctive progressive pregnancy disorder, is characterized by the onset of hypertension and proteinuria after 20 weeks of gestation. Placental pathology in the pre-eclamptic phase primarily manifests as reduced infiltration ability of trophoblast cells. Contributing factors include autophagy, metabolic abnormalities, inflammation, and oxidative stress^131–133^. Studies have indicated that patients with pre-eclampsia exhibit lower expression of SIRT1 in placental and serum samples. Additionally, mice models with SIRT1 knockout (SIRT1^+/−^) exhibit typical pre-eclampsia-like symptoms, such as hypertension, proteinuria, fetal growth restriction, renal injury, and narrowing of placenta's labyrinth layer. Therefore, SIRT1 potentially plays a crucial role in the pathogenesis of pre-eclampsia^134^.

### Preclinical study

Intraperitoneal injection of SRT2104 has been found to ameliorate pre-eclampsia-like symptoms caused by SIRT1 downregulation. This intervention improves renal injury, reduces protein concentration in late pregnancy urine, and inhibits of trophoblast cell apoptosis, thereby potentially preventing the progression of pre-eclampsia^135^. However, to establish SRT2104 as a feasible treatment for pre-eclampsia, further exploration through additional animal experiments and clinical trials is necessary.

### Mechanism of action

SIRT1, an essential protein expressed in granulosa cells (GCs) at various stages of mammalian follicular development, including different phases of the human reproductive cycle^136–139^, has been shown to play a crucial role in reproduction. Studies using animal models have highlighted the significance of SIRT1 activity in reproductive system development, evidenced by defects observed in SIRT1 knockout mice^93,140^. A key player in follicular development is the second messenger molecule cyclic adenosine monophosphate (cAMP), plays a crucial role throughout follicular development. High levels of cAMP, which maintains oocytes in a meiotic arrested state within follicular. Importantly, there exists reciprocal relationship between cAMP and SIRT1, whereby cAMP enhances SIRT1 signaling, While SIRT1 ensures the maintenance of high cAMP levels^141,142^.

The study by Szymanska et al. first shed light on the impact of SRT2104 on GCs. SRT2104 was found to enhance cAMP production in the culture medium of human granulosa-lutein cells by 1.4-fold. Moreover, when combined with human chorionicadotrop (hCG), SRT2104 further increased cAMP generation by 2.2-fold^136^. Evaluation of the effects of SRT2104 on the expression of key genes in human granulosa-lutein cells demonstrated a significant enhancement in ovulation and angiogenesis-related gene expression^143^. In addition, SRT2104 was to inhibit endothelin-2 (EDN2)^144^. Based on the mutual regulatory interactions between SIRT1 and the cAMP signaling pathway, it is reasonable to attribute the similar effects of SRT2104 and hCG on ovulation and angiogenesis-related genes to elevated intracellular cAMP levels^145^.

## SRT2104 in β-Thalassemia

β-Thalassemia, also known as beta-globin deficiency anemia, is an inherited blood disorder that is characterized by a reduction or absence in the synthesis of the β-globin chain in hemoglobin^146^. The therapeutic induction of fetal hemoglobin (HbF) presents a promising avenue for ameliorating clinical symptoms in β-thalassemia patients. Enhancing the expression of the γ-globin gene (HBG), a key component of HbF, is the primary strategy for increasing HbF. In this context, SIRT1, a recently discovered HBG inducer, has gained significant attention^147,148^.

### Mechanism of action

Studies have shown that SRT2104, with the ability to activate SIRT1, effectively reactivate silenced HBG in erythrocytes in a dose-dependent manner. Treatment with SRT2104 at concentrations of 500 nM and 2 μM resulted in an average of 2.6-fold and 4.1-fold increase in HBG mRNA, respectively. Moreover, treatment with 2 μM SRT2104 led to a five-fold increase in HBG protein levels compared to the control group. It is noteworthy that SRT2104 may regulate the HBG production through transcriptional reprogramming mechanisms, facilitated by SIRT1's ability to promote long-range chromatin looping from the locus control region (LCR) to the HBG promoter. This process inhibits the expression of HBG transcriptional factors and indirectly enhances histone acetylation within the HBG promoter, consequently activating HBG expression^149^.

Although the exact mechanism through which SRT2104 selectively activates SIRT1 to modulate HBG transcription remains to be fully elucidated, existing evidence suggests that SIRT1 could be a viable strategy for treating β-thalassemia. In this context, SRT2104 emerges as a potentially promising new therapeutic agent for future β-thalassemia treatments.

## Conclusion and future perspectives

**Figure 3:**
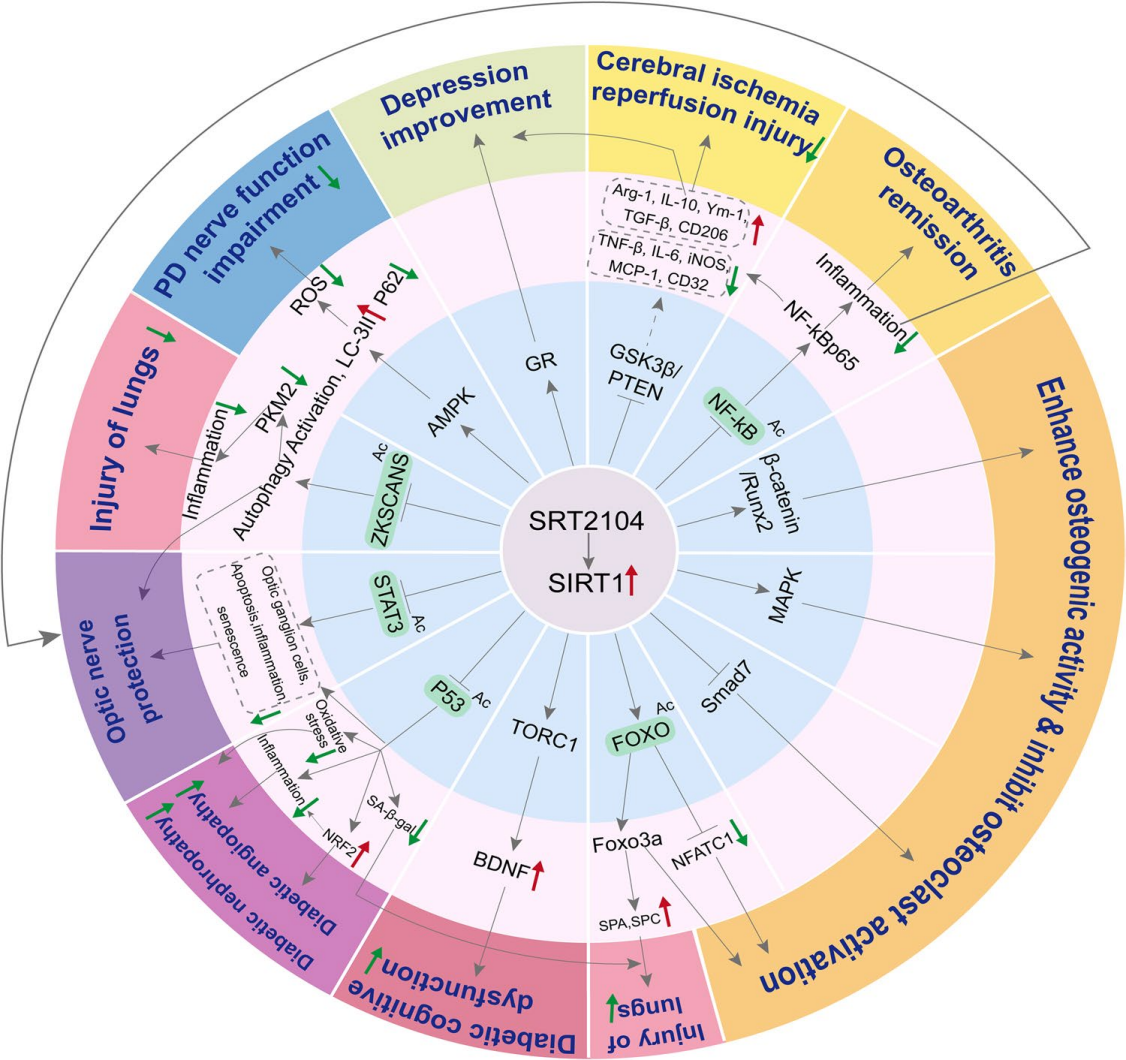
Schematic representation of the molecular mechanisms mediated by SRT2104. SRT2104, through the activation of SIRT1, orchestrates a network of molecular pathways, including P53, STAT3, ZKSCANS, AMPK, GR, GSK3β/PTEN, NF-κB, β-catenin/Runx2, MAPK, Smad7, FOXO, and TORC1. These interactions collectively contribute to the amelioration of conditions such as lung injury, diabetic vascular complications, diabetic nephropathy, cognitive impairments associated with diabetes, musculoskeletal disorders, brain ischemia–reperfusion injury, Parkinson's disease (PD) neurodegeneration, and optic nerve damage. Key: "→" denotes activation or promotion, "⊥" indicates inhibition, "red arrow up" signifies upregulation, "green arrow down" signifies downregulation, and "Ac" refers to deacetylation.

**Figure 4:**
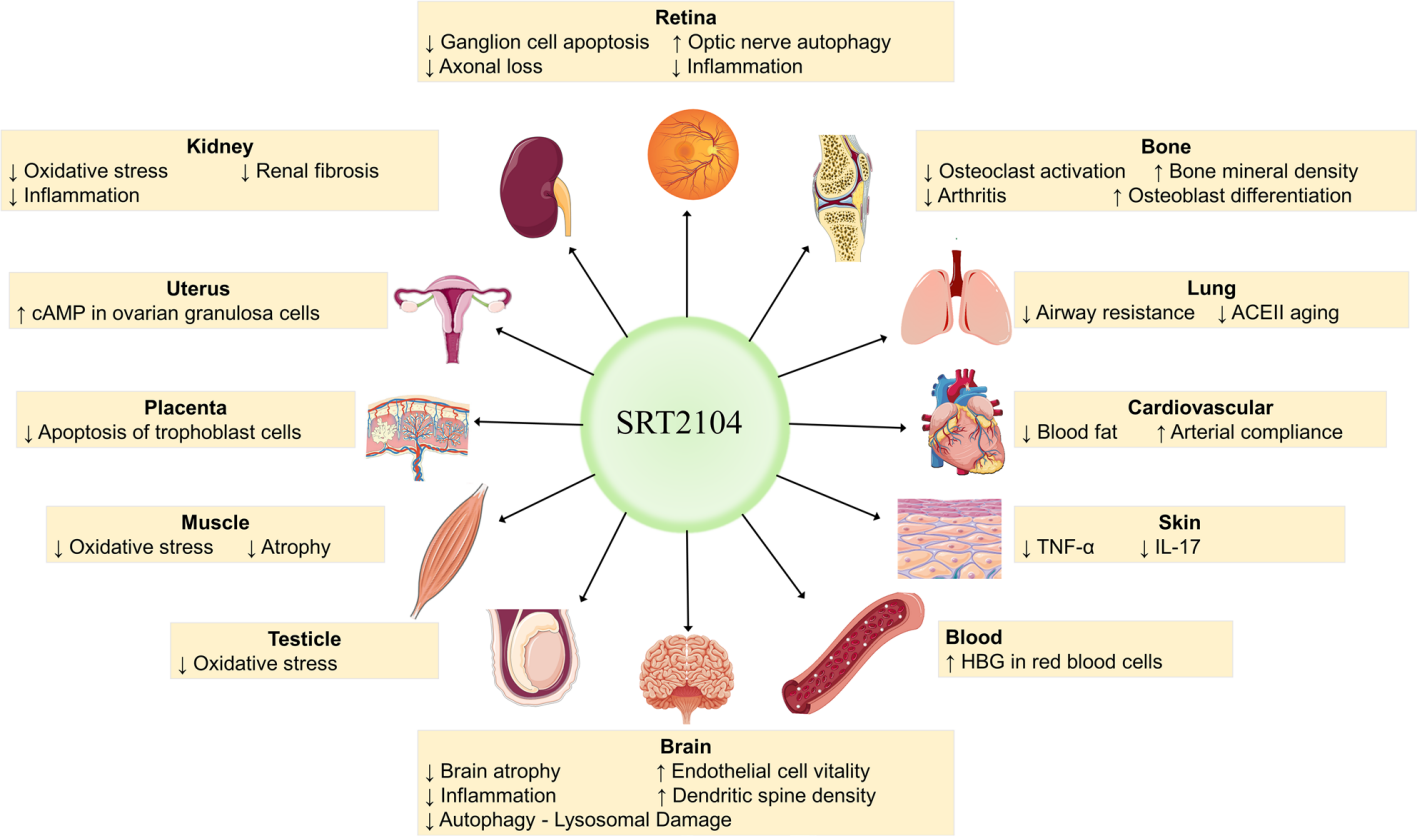
Overview of the potential effects of SRT2104 on various organ systems. The diagram illustrates the compound's impact on key physiological processes across different tissues. “↓”denotes a decrease or impairment, while “↑” signifies an increase or enhancement. This figure summarizes the multifaceted pharmacological actions of SRT2104, suggesting its potential therapeutic applications.

SIRT1 has recently emerged as a promising target for drug design, demonstrating significant potential for therapeutic development. SRT2104, as a novel, efficient, and specific SIRT1 activator, has shown promising tolerability in animal models and clinical trials. This review has methodically examined SRT2104's application in disease treatment, including clinical trial progress, preclinical research advances, and the elucidation of its molecular mechanisms (Fig. 3). Consequently, we have synthesized the diverse impacts of SRT2104 on various diseases (Fig. 4), aligning with our goal to assess SRT2104's therapeutic potential and to clarify its molecular interactions.

SRT2104 has demonstrated favorable therapeutic effects across various conditions such as neurodegenerative diseases, cardiovascular diseases, depression, psoriasis, and others. Its therapeutic efficacy is primarily mediated through the regulation of energy homeostasis, antioxidant stress response, modulation of inflammatory reactions, and autophagy pathways. The idea of a single drug treating multiple diseases, once seen as far-fetched, now appears attainable due to advancements in molecular biology and extensive reach.

However, the application of SRT2104 is still confined to basic research, with a substantial gap remaining between potential and clinical practice. In-depth studies focusing on SRT2104's role in disease treatment and its exact mechanisms are essential for significant clinical breakthroughs. Biomarkers could play a crucial role in monitoring the effectiveness of SRT2104 and tailoring treatment approaches to individual patients. By identifying specific biomarkers associated with the therapeutic response to SRT2104, researchers and clinicians can better understand the drug's impact and optimize treatment regimens. This approach not only promises to enhance the efficacy of SRT2104 but also aids in advancing personalized medicine in the context of the diseases being treated. Nonetheless, it is important to recognize that the journey from drug development to clinical application is arduous and requires time and careful exploration.

Notably, there is a discrepancy between results from cellular/animal models and clinical trials. These discrepancies may arise from varying methodologies used in models versus human diseases. It is crucial to employ models that more accurately mimic human diseases.

For distinct diseases, choosing appropriate administration methods, like targeted drug delivery, is very important to enhance drug efficacy. The duration of therapy is a critical factor that can significantly impact the drug's efficacy. Future research should not only focus on the efficacy and safety of SRT2104 but also on optimizing treatment protocols, including the determination of the most effective treatment durations for different conditions. This focus is vital to ensure that patients receive the maximum benefit from the drug, with an emphasis on balancing efficacy and minimizing potential side effects. By exploring and establishing optimal treatment durations and delivery methods, the therapeutic application of SRT2104 can be more precisely tailored to individual patient needs and disease specifics, thereby enhancing overall treatment outcomes.

Existing clinical trials on SRT2104 are limited in scale and scope, with some relying on subjective measures of therapeutic effectiveness, which challenges the reliability of their conclusions. Thus, conducting comprehensive, standardized, multicenter, large-scale clinical trials is crucial to confirm SRT2104's effectiveness and safety. Furthermore, exploring SRT2104's role in other diseases and deepening our understanding of its action mechanisms will be invaluable. These efforts will pave the way for a broader development and application of SRT2104, addressing the key areas and questions identified in this review.

## Data Availability

The datasets used and analysed during the current study available from the corresponding author on reasonable request.
